# Association of Genetic Variants of *RANK*, *RANKL*, and *OPG* with Ankylosing Spondylitis Clinical Features in Taiwanese

**DOI:** 10.1155/2019/8029863

**Published:** 2019-03-20

**Authors:** Chin-Man Wang, Shu-Chun Tsai, Jing-Chi Lin, Yeong-Jian Jan Wu, Jianming Wu, Ji-Yih Chen

**Affiliations:** ^1^Department of Rehabilitation, Chang Gung Memorial Hospital, College of Medicine, Chang Gung University, Taiwan; ^2^The Genomics Research Center, Academia Sinica, 128 Academia Road, Section 2 Nankang, Taipei 115, Taiwan; ^3^Attending Physician, Department of Medicine, Division of Allergy, Immunology and Rheumatology, Chang Gung Memorial Hospital, College of Medicine, Chang Gung University, Taiwan; ^4^Associate Professor, Department of Veterinary and Biomedical Sciences, Department of Medicine, University of Minnesota, USA

## Abstract

Ankylosing spondylitis (AS) is a chronic inflammatory disease that leads to spinal ankylosis. The receptor activator of the nuclear factor-kappa (RANK), RANK ligand, and osteoprotegerin (OPG) (RANK/RANKL/OPG) pathway plays critical roles in bone metabolism and the immune system. The current study was aimed at investigating whether six single-nucleotide polymorphisms (SNPs) within the *RANK*, *RANKL*, and *OPG* genes essential for bone homeostasis are associated with AS. Genotype distributions, allele and haplotype frequencies, were compared between 1120 AS patients and 1435 healthy controls and among AS patients with stratification by syndesmophyte formation, onset age, and HLA-B27 positivity. We found that *RANKL* SNPs were associated with AS syndesmophyte formation. Notably, the *RANKL* SNP haplotype rs7984870C/rs9533155G/rs9525641C was negatively associated with AS susceptibility and appeared to protect against syndesmophyte formation in AS. Functionally, *RANKL* promoter SNPs (rs9525641 C/T and rs9533155 G/C) affected DNA-protein complex formation and promoter activity in promoter reporter analyses. The *OPG* SNP haplotype rs2073618G/rs3102735T was significantly associated with HLA-B27 negativity in AS patients. Furthermore, AS patients with syndesmophyte formation had significantly lower levels of soluble RANKL levels than those without syndesmophyte formation. Our data suggested a role for *RANKL* in AS susceptibility and severity.

## 1. Introduction

Ankylosing spondylitis (AS) is a chronic inflammatory disease primarily affecting the axial bones, leading to spinal ankylosis. Multiple factors including genetic compositions, infectious agents, and immune response dysfunctions contribute to the development of AS in individuals. Most AS patients are positive for the human leukocyte antigen (HLA) B27. Nevertheless, overwhelming majority of individuals who are positive for HLA-B27 do not have AS, and therefore, HLA-B27 positivity only accounts for a small fraction of the AS heritability [1]. Moreover, there is high interindividual variability in the rate of AS disease progression based on radiographic assessment of syndesmophyte formation [2–4].

Bone morphogenesis and remodeling are tightly regulated processes characterized by continuous bone synthesis and resorption, which are controlled by functions of osteoblasts and osteoclasts and intercellular cell-cell interactions [5–7]. A proper balance between bone resorption and synthesis is essential to maintain bone mechanical strength and structure [8]. In AS patients, chronic inflammation and osteogenesis break the balance between bone resorption and synthesis, resulting in structural bone damage, spinal immobility, and significant functional impairment [9–11]. The osteogenesis in AS patients is also accompanied by bone loss, causing systemic osteopenia or osteoporosis and spinal damage [12–15]. The excessive resorption of trabecular bone and the synthesis of new cortical bone at sites of inflammation are manifested as spinal ankylosis with fusion of sacroiliac joints [16].

Cytokines regulating osteoclast formation and function likely contribute to hyperactive osteoclastogenesis in AS patients. The receptor activator of nuclear factor-kappa ligand (RANKL) is a membrane-bound TNF-related factor expressed by osteoblasts, stromal cells, fibroblasts, and activated T cells [17–19]. Osteoclast differentiation requires the interaction between RANK expressed on osteoclast precursors and RANKL expressed on osteoblasts and stromal cells [20–24]. RANKL activates several signalling pathways to fine-tune bone homeostasis through controlling osteoclast development and bone loss [25]. RANKL upregulates the activity of the transcription factor nuclear factor-activated T cell c1 (NFATc1) to tightly regulate osteoclast differentiation [26]. The MHC class II transactivator and SWAP-70-like adapter of T cells (SLAT) decrease RANKL-mediated osteoclast differentiation by downregulating NFATc1 [27, 28]. Therefore, RANKL promotes osteoclast differentiation, maturation, and activation and contributes to osteoporosis by promoting bone resorption.

Osteoprotegerin (OPG) is a soluble RANKL “decoy” receptor that inhibits RANKL function, thereby decreasing osteoclast development [22, 29]. The increased synovial expressions of RANKL and OPG in peripheral spondylarthritis were unlikely associated with the degree of systemic and local inflammation in most patients [30]. However, patients that responded well to TNF alpha blockade therapy had decreased RANKL expression in the intimal lining layer [30]. Thus, the RANK/RANKL/OPG axis appears to affect the pathogenesis and clinical outcome of some patients with AS. Genetic variation at the *OPG* locus was reported to associate with the clinical features of AS [31]. Nevertheless, it remained unclear whether *RANKL* single-nucleotide polymorphisms (SNPs) have a role in AS. The purpose of current study was to investigate whether genetic variants of *RANK*, *RANKL*, and *OPG* are associated with AS disease susceptibility and clinical manifestations.

## 2. Material and Methods

### 2.1. Study Subjects

AS patients (926 males and 194 females) who fulfilled the 1984 revised New York diagnostic criteria for AS [32] were recruited at the Chang Gung Memorial Hospital (a 3600-bed medical center and university hospital). Two rheumatology specialists independently evaluated syndesmophyte formation and graded the severity of AS according to the modified Stoke's Ankylosing Spondylitis Spinal Score (mSASSS) through examining radiographs of cervical, thoracic, and lumbar spines [33, 34]. Based on radiography, AS patients were placed into three groups: no syndesmophytes, mSASSS = 0 (group 1), fewer than 4 syndesmophytes, mSASSS < 24 (group 2), and 4 or more syndesmophytes, mSASSS ≥ 24 (group 3) [34]. The rare disagreements of these evaluations were resolved by consultations between physicians. The presence or absence of HLA-B27 was determined by flow cytometry and/or PCR assays. A total of 1435 healthy blood donors (798 males and 637 females, age range: 18 to 64 years, mean age: 42.89 ± 12.81) were recruited as normal healthy controls. The study was carried out in accordance with the relevant guidelines and regulations. All experimental protocols were approved by the ethics committee of Chang Gung Memorial Hospital, and informed consent was obtained from all subjects.

### 2.2. Nucleic Acid Isolation

Anticoagulated peripheral blood was obtained from AS patients and the comparison group (healthy subjects). Genomic DNA was isolated from anticoagulated peripheral blood using the Puregene DNA isolation kit (Gentra Systems, Minneapolis, MN, USA) as previously described [35].

### 2.3. RANK, RANKL, and OPG SNP Analyses

Matrix-assisted laser desorption ionization time-of-flight (MALDI-TOF) mass spectrometry was used to genotype the *RANKL* promoter SNPs as previously described [35]. Briefly, the DNA fragment-containing *RANKL* proximal promoter was amplified using the forward primer nucleotide sequence 5′ to 3′ F: TTTTAAAAAGCCCTAGCAAGGT and the reverse primer nucleotide sequence 5′ to 3′ F: TTGTCTGCGGCCAACTC in 96-well PCR plates with 15 ng of genomic DNA. The 865 bp PCR products were automatically purified on the MALDI-TOF MAP II/8 Robotic Platform using the Genopure DS Magnetic Bead DNA Purification kit (Bruker Daltonics). The purified DNA fragment was subsequently used for the PCR-based SNP detection assays with a SNP-specific detection primer and a termination mix. The SNP allele-specific products purified with the Genopure Oligo Magnetic Bead DNA Purification kit (Bruker Daltonics) were automatically spotted onto the 384-well AnchorChip plate and analyzed by AutoFlex mass spectrometry. Genotype data from MALDI-TOF analyses completely matched with the results of Sanger sequencing analysis in 39 samples using the BigDye terminator sequencing kit on an ABI 3100 sequencer (Applied Biosystems), confirming the reliability of MALDI-TOF SNP assays.

*RANK* and *OPG* SNPs were genotyped with the TaqMan SNP Made-to-Order allelic discrimination assays (Applied Biosystems). Allele-specific probes labeled with a fluorescent dye (FAM or VIC) were used in TaqMan SNP analyses on a real-time PCR thermocycler (Applied Biosystems). Genotypes were determined using TaqMan Genotyper software (Applied Biosystems) according to the vendor's instructions.

#### 2.3.1. Gene Cloning and Plasmid Preparation

The *RANKL* promoter fragment was amplified with PCR using human genomic DNA. The purified *RANKL* promoter region was cloned into the pGL4.20 vector (Promega) at the XhoI and HindIII sites. The luciferase reporter plasmids were prepared by using the Geneaid mini plasmid kit (Geneaid).

#### 2.3.2. Site-Directed Mutagenesis of the RANKL Promoter

The confirmed plasmid of the *RANKL* promoter was mutated at three sites by a quick-change Tm XL site-directed mutagenesis kit (Stratagene, Agilent). The nucleotide sequences of the cloned constructs were confirmed by direct sequencing from both directions on an ABI 377 Sequencer with ABI BigDye Terminator Cycle Sequencing Kit.

#### 2.3.3. Electrophoretic Mobility Shift Assay (EMSA)

Nuclear extracts of human fetal osteoblastic (hFOB) 1.19 cells were harvested as previously described [36]. LightShift Chemiluminescent EMSA kit (Thermo Scientific) was used for EMSA analyses according to the manufacturer's instructions. Briefly, both forward and reverse strands of polynucleotides were labeled with biotin at the 5′ ends and then annealed to form a double-strand probe. Four probes (−693C, −693T, −290T, and −290C) covering 2 regions of the *RANKL* promoter were used in this assay. The −693C double-strand DNA probe has the nucleotide sequence 5′ to 3′ F: TGTTGGGTGAGCCCT***C***CTCGGATGCTTGCT while the −693G double-strand DNA probe has the nucleotide sequence 5′ to 3′ F: TGTTGGGTGAGCCCT***G***CTCGGATGCTTGCT. The −290T double-strand DNA probe has the nucleotide sequence 5′ to 3′ F: CCTCTGCGTCTTC***T***TTAACCCATCTCTTGG while the −290C double strand DNA probe has the nucleotide sequence 5′ to 3′ F: CCTCTGCGTCTTC***C***TTAACCCATCTCTTGG. The binding reaction was performed in a total volume of 20 *μ*L with 6 *μ*g of nuclear extracts, 2.5 nM biotin-labeled probe, 1 *μ*g poly(dI-dC), 2.5% glycerol, and 0.05% Nonidet P-40 (NP40) in 1x binding buffer. For competition experiments, 500 nM of unlabeled probes was added. For antibody supershift experiments, 2 *μ*g of anti-Sp1 antibody (Sigma) or normal mouse IgG (Millipore) was added. All reaction mixtures were incubated at room temperature for 30 min, electrophoresed in 6% native polyacrylamide gels with Tris-borate buffer (90 mM Tris, 90 mM boric acid, and 2 mM EDTA), and then transferred to Hybond-N membranes (Amersham Biosciences). The membranes were UV crosslinked and blocked, and the signals were measured with the Chemiluminescent Nucleic Acid Detection Module (Pierce) according to the manufacturer's instructions.

#### 2.3.4. Luciferase Promoter Reporter Assay

hFOB 1.19 cells seeded in 12-well plates at 50% confluence were transfected with 0.8 *μ*g of *RANKL* promoter reporter plasmid DNA plus 0.2 *μ*g of internal control pEGFP-C1 (Promega) plasmid DNA using the TransIT-2020 transfection reagent (Mirus). Cells were harvested 72 h after transfection and subsequently lysed with Passive Lysis Buffer (Promega). Bright-Glo Luciferase Assay reagent (Promega) was used to determine luciferase activity and GFP fluorescence values. The relative fold of reporter activity normalized by GFP intensity represented the ratios of promoter reporter luciferase values versus the pGL4 control luciferase value (Supplemental Figure 1).

#### 2.3.5. Soluble RANKL Assay

The total soluble RANKL (sRANKL) ELISA kit (Enzo Life Sciences; catalog: ALX-850-019-KI01) was used to measure serum RANKL levels in the sera of AS patients by following the manufacturer's instructions.

#### 2.3.6. Statistical Analysis

Promoter activity data are presented as means and standard errors of the means (SEM). The 2-tailed *t*-test was used to compare the levels of two polymorphic human *RANKL* luciferase reporter constructs. The correlation between different parameters was assessed by linear regression analysis and Pearson's coefficient of correlation. Three chi-squared tests (the genotype test, allele test, and Cochran-Armitage trend test) were performed to analyze the associations of SNPs with AS disease susceptibility and phenotypes. Based on the risk allele identified, *p* values, odds ratios (ORs), and 95% confidence interval (CIs) were then calculated. To account for the multiple testing corrections, the FDR-corrected *p* values were generated by using false discovery rate (FDR) correction using the modified version of FDR programmed in QVALUE software (http://genomics.princeton.edu/storeylab/qvalue/). Linkage disequilibrium (LD) between marker loci was measured, and haplotype blocks were constructed using Haploview 4.2 (Broad Institute, Cambridge, MA, USA; http://www.broad.mit.edu/mpg/haploview). The SAS HAPLOTYPE procedure was used to infer haplotype information and estimation of frequencies. Association of the estimated haplotypes and disease status was tested by logistic regression models. For the markers within the same haplotype block, we used disease status (case vs control) and clinical characteristics (early age onset, HLA B27 positivity, and syndesmophyte formation) as traits and tested for the haplotype-trait association utilizing the haplo.stat SAS HAPLOTYPE procedure. Mann-Whitney *U* tests were used to analyze the serum sRANKL levels among AS patients using GraphPad Prism 6.0 (GraphPad, La Jolla, CA, USA). The 5% level of significance for *p* values was used for all analyses.

## 3. Results

### 3.1. Clinical Characteristics of Patients with AS

Among 1120 Taiwanese AS patients (926 males and 194 females), 297 patients had disease onset ages of ≤16 years while 823 patients had the onset ages between 17 and 60 years. Within the AS cohort, 1021 patients (91.2%) were positive for HLA-B27 and 485 patients (43.3%) were positive for syndesmophyte formation based on spinal X-ray analysis (146 patients with mSASSS being less than 24 and 339 patients with mSASSS of being 24 or more).

### 3.2. Association of SNPs of *RANK*, *RANKL*, and *OPG* with AS Susceptibility

We carried out genetic analyses to investigate whether *RANK*, *RANKL*, and *OPG* genes are involved in AS susceptibility. The *RANK* SNP (rs1805034), three *RANKL* SNPs (rs7984870, rs9525641, and rs9533155), and two *OPG* SNPs (rs2073618 and rs3102735) were genotyped in 1120 AS patients and 1435 healthy controls. The *RANKL* SNP rs7984870G allele (trend test, *p* = 0.0413 with 100000 permutations), rs9533155C allele (trend test, *p* = 0.048), and *RANKL* SNP rs9525641T (trend test, *p* = 0.0297) tended to be enriched in AS patients (Table 1). However, no significant associations were observed between all six SNPs and AS susceptibility after the corrections for multiple hypothesis testing. In addition, no significant deviations from the Hardy-Weinberg equilibrium in the distributions of genotypes and alleles were observed for all SNPs in AS patients and normal controls, indicating that individual genetic variants of *RANK*, *RANKL*, and *OPG* may not have an effect on AS susceptibility.

### 3.3. Association of *RANKL*, *RANK*, and *OPG* SNPs with Clinical Characteristics of AS Patients

AS is a polygenic disease manifested with diverse clinical symptoms and disease severity. Accordingly, we carried out genetic analyses of *RANKL*, *RANK*, and *OPG* SNPs by stratifying AS patients based on HLA-B27 positivity and clinical characteristics. As shown in Table 2, *RANKL* SNP rs7984870G allele (trend test *p* = 0.0083; *p*_FDR_ = 0.022), rs9525641T allele (trend test *p* = 0.0064; *p*_FDR_ = 0.022), and rs9533155C allele (trend test: *p* = 0.011; *p*_FDR_ = 0.022) were significantly enriched in AS patients positive for syndesmophyte formation as compared to normal controls. The *RANKL* rs7984870G allele carriers (genotypes GG + CG vs CC; *p*_FDR_ = 0.0325, OR = 1.42, 95% CI = 1.08-1.86), the rs9525641T allele carriers of (TT + CT vs CC; *p*_FDR_ = 0.0325, OR = 1.40, 95% CI = 1.07-1.84), and rs9533155C allele carriers (CC + GC vs GG; *p*_FDR_ = 0.0325, OR = 1.41, 95% CI = 1.07-1.86) were also significantly increased in the AS patients with syndesmophyte formation as compared to normal controls. In addition, the *RANKL* SNP rs7984870G allele (trend test: *p* = 0.0269; *p*_FDR_ = 0.0638), rs9525641T allele (trend test: *p* = 0.0206; *p*_FDR_ = 0.0638), and rs9525641C allele (trend test: *p* = 0.0319; *p*_FDR_ = 0.0638) were significantly associated with HLA-B27 positivity (Table 3). No significant associations were observed between clinical characteristics and SNPs of *RANK* and *OPG* among AS patients.

### 3.4. Association of the *RANKL* Promoter and *OPG* SNP Haplotypes with AS Susceptibility and Clinical Manifestations

Since *RANKL* promoter SNP haplotypes may differentially affect promoter functions, we carried out haplotype analyses to assess the association of *RANKL* SNP haplotypes with AS susceptibility and syndesmophyte formation. Two common *RANKL* SNP haplotypes (rs7984870C/rs9533155G/rs9525641C and rs7984870G/rs9533155C/rs9525641T) and several rare haplotypes with frequencies less than 0.05 were deduced. We found that the *RANKL* rs7984870C/rs9533155G/rs9525641C (or *RANKL* CGC) haplotype was significantly decreased in AS patients as compared to normal controls (AS: 41.83%, control: 45.88%; adjusted *p* = 0.0081, OR = 0.85, 95% CI = 0.76‐0.96), suggesting that the *RANKL* CGC haplotype may have a protection role against the development of AS (Table 4). In addition, the frequency of the *RANKL* CGC haplotype appeared to decrease in AS patients with syndesmophytes as compared to AS patients negative for syndesmophytes (AS syndesmophyte positive: 39.71%, AS syndesmophyte negative: 43.45%; adjusted *p* = 0.0725, OR = 0.85, 95% CI = 0.76-1.01) (Supplemental Table 2). However, *RANKL* SNP haplotypes were not associated with either HLA-B27 positivity or early age of onset among AS patients (Supplemental Tables 3 and 4). Two *OPG* SNPs formed four SNP haplotypes on chromosome 8. No associations were observed between four major *OPG* haplotypes and AS susceptibility (Supplemental Table 5), syndesmophyte formation (Supplemental Table 6), and age of disease onset (Supplemental Table 7). Nevertheless, the *OPG* rs2073618G/rs3102735T (or *OPG* GT) haplotype was significantly enriched in HLA-B27-negative AS patients (*p* = 0.0373, OR = 0.7; 95% CI = 0.51-0.98) as compared to AS patients positive for HLA-B27 (Supplemental Table 8). Taken together, the *RANKL* SNP haplotype rs7984870C/rs9533155G/rs9525641C seems to associate with the low risks for AS and AS syndesmophyte formation. On the other hand, the *OPG* SNP haplotype rs2073618G/rs3102735T may associate with HLA-B27 negativity in AS patients.

### 3.5. *RANKL* Promoter SNPs Affect Promoter Functions

**Figure 1:**
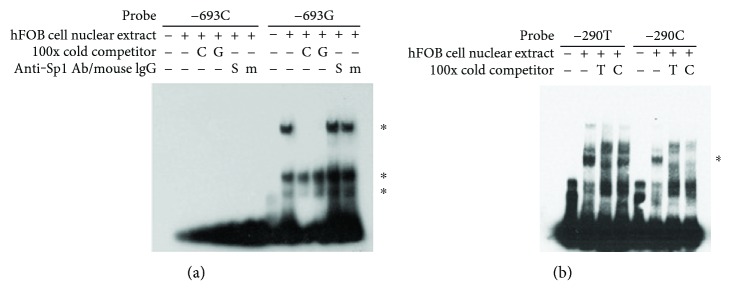
*RANKL* promoter probes containing SNPs bind nuclear proteins with different affinities. A nuclear extract of hFOB cells was incubated with biotin-labeled probes in the presence or absence of a 100-fold excess of unlabeled probes, 2 *μ*g anti-Sp1 antibody (S), or mouse IgG (m). After 30 min, the reaction mixture was analyzed by EMSA. (a) Nuclear proteins formed 3 complexes with the -693G probe, but none with the -693C probe. (b) Nuclear proteins and DNA probes formed more complexes with the −290T probe than with the -290C probe at the indicated region. Asterisks indicate protein-DNA complexes.

Since *RANKL* SNP haplotypes were significantly associated with risk for AS. We attempted to identify novel SNPs in the *RANKL* proximal promoter region. Sequencing analyses of a 1 kb *RANKL* proximal promoter region revealed three SNPs: −693G/C (rs9533155), −643C/T (rs9533156), and −290C/T (rs9525641). Results of transcription factor-searching software based on the matrix of nucleotide sequence identity indicate that the SNPs −693G/C and −290C/T may sit at a putative specificity protein 1 (Sp1) transcription factor-binding site. We carried out EMSA analyses to examine the effect of those *RANKL* SNPs on the binding to transcription factors. As shown in Figure 1, the probe containing the -693G allele could form three DNA-protein complexes while none of the complexes could be formed with the probe containing -693C (lanes 2 and 8, Figure 1(a)). Anti-Sp1 antibody (S) failed to cause supershifts or the disruption of three DNA-protein complexes (lane 11, Figure 1(a)), suggesting that Sp1 is not included in those DNA-protein complexes. A 200-fold excess of −693C (C) or −693G (G) unlabeled probes effectively blocked the formation of the high-molecular weight complex but only slightly inhibited the formation of two low-molecular weight complexes (lanes 9 and 10, Figure 1(a)). Thus, even though the −693C probe failed to form the tight DNA-protein complexes, an excess of the -693C probe that may have low affinity for nuclear proteins had the ability to interfere the binding of the -693G probe to nuclear proteins.  Figure 1(b) showed that the −290T probe appeared to form more DNA-nuclear protein complexes than did the −290C probe.

Subsequently, we carried out promoter reporter assays to determine the activity of the three most common *RANKL* SNP haplotypes (-693C/−643T/−290T or CTT, −693C/−643T/−290C or CTC, and −693G/−643C/−290C or GCC). We found that all three *RANKL* promoter reporters (CTT, CTC, and GCC) yielded significantly lower luciferase activities than did the pGL4 vector control (Supplemental Figure 1), suggesting that the proximal *RANKL* promoter region contains strong repressors. Interestingly, the *RANKL* CTC promoter reporter had the lowest activity, suggesting that the CTC haplotype allele may constitute the strongest repressor. Notably, the common haplotypes CTT and GCC had the similar promoter activity. Our data indicate that proximal *RANKL* promoter SNPs may affect *RANKL* promoter function.

### 3.6. Association of sRANKL Levels with AS Syndesmophyte Formation

**Figure 2:**
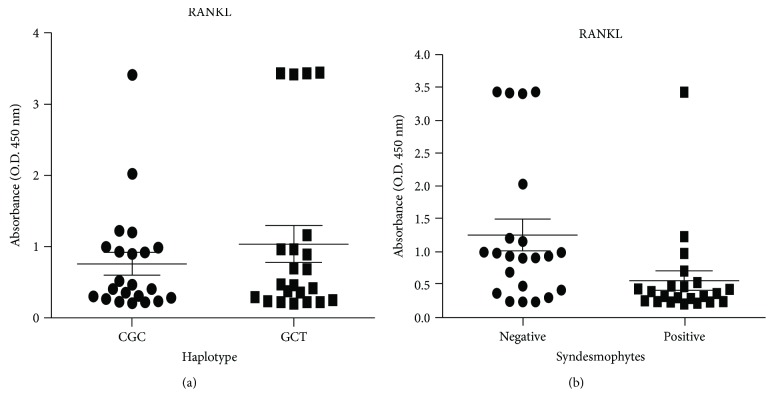
Total serum levels of soluble receptor activator nuclear factor kappa B ligand (sRANKL) in patients with ankylosing spondylitis (AS). (a) Concentrations of serum sRANKL in homozygous carriers of the RANKL CGC haplotype (rs7984870, rs9533155, and rs9525641) compared to those in homozygous carriers of the RANKL GCT haplotype. (b) Concentrations of serum sRANKL in AS with syndesmophyte formation compared with those without syndesmophyte formation (*p* = 0.0054, by Mann-Whitney *U* test).

Our genetic analyses indicate that *RANKL* may be involved in the development of AS. Subsequently, we measured soluble RANKL (sRANKL) levels in the serum of AS patients to determine whether sRANKL levels are associated with *RANKL* SNP haplotypes and syndesmophyte formation. As shown in Figure 2, sRANKL levels were not significantly different between two common *RANKL* haplotypes (CGC and GCT). However, AS patients with syndesmophyte formation had significantly lower sRANKL serum levels (*N* = 22, mean ± SEM = 0.54573 ± 0.14767) than did those without syndesmophyte formation (*N* = 22; mean ± SEM = 1.2488 ± 0.24056; *p* = 0.0054). Our data indicate that sRANKL may block syndesmophyte formation in AS patients.

## 4. Discussion

The bone is a critical target in the development of AS. The disruption of normal bone remodeling in AS patients is characterized by local and systemic bone loss and subsequent new bone formation [9]. In the current genetic study, the *RANKL* SNP haplotype rs7984870C/rs9533155G/rs9525641C was identified as a protecting factor against the development of AS in large cohorts of Taiwanese AS patients and healthy controls. More importantly, we found that the low serum sRANKL levels were significantly associated with syndesmophyte formation in AS patients, suggesting a protective role of sRANKL against syndesmophyte formation. Our data provided new insights into the functional roles of the RANK/RANKL/OPG axis in the pathogenesis of AS. A limitation of the current study is that the syndesmophyte formation was considered as an established manifestation. We should be cautious in interpreting the genetic association between SNPs and syndesmophyte formation.

The progressive joint destruction and bone mineral degradation in AS patients are due in part to the activity of numerous proteolytic enzymes synthesized by osteoclasts [37]. Osteoclastogenesis is significantly increased in AS patients with sacroiliac joint ankyloses. In addition, AS patients had high levels of serum sRANKL and OPG, which may relate to the disease progression and clinical outcomes [38]. The production of OPG might reflect systemic inflammation as OPG levels are associated with poor physical mobility in AS patients [38]. The sRANKL levels and the sRANKL/OPG ratio were found to correlate with bone mineral density (BMD) and radiological changes in AS patients [39]. Taken together, RANKL and OPG may be involved in the pathogenesis of AS.

Several studies have been carried out to investigate the effect of genetic variations of those genes on osteoclast-related conditions, such as osteoporosis [40, 41], and autoimmune diseases [42–47]. The *RANKL* SNP rs2277438 reportedly contributes to the radiographic progression of RA in a Japanese population [43]. In addition, carriers of the *RANKL* SNP rs7984870-CC genotype had twofold higher plasma levels of sRANKL and an earlier age of RA onset, particularly in those patients with antibodies against cyclic citrullinated peptide (CCP) and rheumatoid factor (RF) [48, 49]. RF-positive RA patients carrying the *RANKL* SNP rs7984870-CC genotype also had significantly elevated *RANKL* mRNA expression in the activated T cells. Therefore, the *RANKL* SNP rs7984870C>G located in the distal *RANKL* promoter (−1816) may play a key role in disease pathogenesis through regulating RANKL production.

The *RANKL* proximal promoter (1 kb) interacts with transcription factors such as heat shock proteins, vitamin D3, CCAAT/enhancer-binding protein beta (CEBP beta), E2F1 (E2F transcription factor 1), SP1 (specificity protein 1), SP3 (Sp3 transcription factor), and core-binding factor a1 (Cbfa1), which affect the expression of *RANKL* [50–56]. The deletion analysis revealed the region between nucleotide positions −300 to −1000 contains the *RANKL* promoter repressor, where the SNPs −693G>C (rs9533155) and −643C>T (rs9525641) are located [54]. In the current study, we found that *RANKL* SNP −693G>C and -290C>T significantly affect the formation of DNA-protein complexes. Nevertheless, we failed to identify specific transcription factors that bind to the regions containing *RANKL* SNPs in EMSA analyses. Our promoter reporter analysis confirmed that the *RANKL* SNPs −693G>C and −290C>T are within a promoter repressor as the *RANKL* promoter SNP haplotypes differently suppressed the promoter activities, suggesting that *RANKL* promoter SNPs may be functional.

Interestingly, we found that the common *RANKL* promoter rs7984870C/rs9525641G/rs9525641C (CGC) haplotype with high-activity rs7984870C allele was associated with the protection against the development of AS. In particular, the common *RANKL* promoter CGC haplotype allele seems to protect against syndesmophyte formation. However, those serum sRANKL levels of AS patients were not associated with *RANKL* promoter haplotypes (Figure 2(a)). We speculate that functional *RANKL* promoter SNPs may influence the *RANKL* gene expression but not the production of sRANKL, which is the cleavage product of a type II membrane protein. The production of sRANKL from membrane RANKL requires the action of proteases [57]. Additionally, we found that sRANKL levels were significantly associated with syndesmophyte formation, suggesting that the production of sRANKL may be influenced by AS disease activity. Our data suggest that the decreased RANKL expression may adversely affect the bone remodeling process in AS patients.

Previous studies showed that an excess of recombinant OPG could block the OPG expression of endothelial cells and macrophages in the synovial lining layer [58]. In addition, the OPG expression in macrophage-type synovial lining cells and endothelial cells was significantly reduced in RA patients with active synovitis and the low levels of OPG were associated with the development of radiologically defined joint erosions in inflamed joints [58]. By contrast, OPG was highly expressed in spondyloarthropathy patients with active synovitis, indicating distinct disease pathways in RA and AS [59]. The *OPG* genetic variation was associated with peripheral arthritis, age of onset, and HLA-B27 positivity in a cohort of AS patients [31]. However, we found a modest association between HLA-B27 positivity and OPG SNPs (rs2073618 and rs3102735). The discrepancies may be explained by different sample sizes and ethnicities of study subjects.

During AS development, the increased peri-inflammatory bone formation is followed by healing of erosions, ossifying enthesitis, and ankylosing of sacroiliac joints and intervertebral connections, eventually biomechanical changes in the spine [15]. The RANKL/RANK/OPG axis is a part of the important pathways controlling bone remodeling in AS. AS patients with syndesmophyte formation had the augmented levels of bone formation markers (bone morphogenetic proteins (BMPs) [60] and wingless-related integration sites (WNTs)) [61] and low concentrations of inhibitors of bone formation (sclerostin and dickkopf-1) [62, 63]. The disruption of the bone remodeling process in AS may involve multiple pathways controlling bone formation and resorption. Further investigations are required to delineate precise pathways involved in the pathogenesis of AS.

## 5. Conclusion

The RANKL/RANK/OPG axis affecting osteoclast differentiation and osteoproliferation may affect AS disease susceptibility and severity.

## Figures and Tables

**Table 1:** Association of *RANK*, *RANKL*, and *OPG* SNPs with AS susceptibility in Taiwanese.

SNP	Risk allele frequency	Genotype frequency (%)			*p*_trend_^∗^	*p*_FDR_	Test for the mode of inheritance unadjusted				Test for the mode of inheritance adjusted for sex			
								*p*	*p*_FDR_	OR (95% CI)		*p*	*p*_FDR_	OR (95% CI)
RANK rs1805034 CT	C	**CC**	**CT**	**TT**			Additive	0.0814	0.122	1.11 (0.99-1.25)	Additive	0.0858	0.1287	1.11 (0.99-1.26)
Case	745 (33.26%)	121 (10.8%)	503 (44.91%)	496 (44.29%)	0.0881	0.1322	CC + CT vs TT	0.0577	0.1387	1.16 (1-1.36)	CC + CT vs TT	0.0651	0.0976	1.17 (0.99-1.37)
Control	881 (30.96%)	142 (9.98%)	597 (41.95%)	684 (48.07%)			CC vs CT + TT	0.4963	0.5239	1.09 (0.85-1.41)	CC vs CT + TT	0.4772	0.5727	1.1 (0.84-1.44)

RANKL rs7984870 CG	G	**CC**	**CG**	**GG**			Additive	0.0407	0.0922	1.12 (1.01-1.25)	Additive	0.0517	0.1274	1.12 (1-1.26)
Case	1203 (53.95%)	235 (21.08%)	557 (49.96%)	323 (28.97%)	0.0413	0.096	GG + CG vs CC	0.0694	0.1387	1.19 (0.99-1.44)	GG + CG vs CC	0.0451	0.0976	1.22 (1.01-1.49)
Control	1456 (51.05%)	344 (24.12%)	708 (49.65%)	374 (26.23%)			GG vs CG + CC	0.1244	0.2488	1.15 (0.96-1.37)	GG vs CG + CC	0.2290	0.4616	1.12 (0.93-1.34)

RANKL rs9525641 CT	T	**CC**	**CT**	**TT**			Additive	0.0301	0.0922	1.13 (1.01-1.26)	Additive	0.0487	0.1274	1.12 (1-1.26)
Case	1204 (54.09%)	235 (21.11%)	552 (49.6%)	326 (29.29%)	0.0297	0.096	TT + CT vs CC	0.0651	0.1387	1.19 (0.99-1.44)	TT + CT vs CC	0.0501	0.0976	1.22 (1-1.48)
Control	1458 (51.01%)	346 (24.21%)	708 (49.55%)	375 (26.24%)			TT vs CT + CC	0.0881	0.2488	1.16 (0.98-1.39)	TT vs CT + CC	0.1976	0.4616	1.13 (0.94-1.35)

RANKL rs9533155 GC	C	**GG**	**GC**	**CC**	0.048	0.096	Additive	0.0461	0.0922	1.12 (1-1.25)	Additive	0.0637	0.1274	1.12 (0.99-1.25)
Case	1241 (55.65%)	219 (19.64%)	551 (49.42%)	345 (30.94%)			CC + GC vs GG	0.1054	0.1581	1.17 (0.97-1.42)	CC + GC vs GG	0.0613	0.0976	1.21 (0.99-1.48)
Control	1508 (52.84%)	318 (22.28%)	710 (49.75%)	399 (27.96%)			CC vs GC + GG	0.1013	0.2488	1.15 (0.97-1.37)	CC vs GC + GG	0.2308	0.4616	1.12 (0.93-1.33)

OPG rs3102735 CT	C	**CC**	**CT**	**TT**	0.5191	0.6229	Additive	0.5074	0.6089	1.05 (0.9-1.23)	Additive	0.2826	0.3391	1.09 (0.93-1.29)
Case	328 (14.72%)	21 (1.89%)	286 (25.67%)	807 (72.44%)			CC + CT vs TT	0.3413	0.393	1.09 (0.91-1.3)	CC + CT vs TT	0.1699	0.2039	1.14 (0.95-1.37)
Control	400 (14.06%)	32 (2.25%)	336 (23.63%)	1054 (74.12%)			CC vs CT + TT	0.5239	0.5239	0.83 (0.48-1.45)	CC vs CT + TT	0.6071	0.6071	0.86 (0.48-1.53)

OPG rs2073618 CG	C	**CC**	**CG**	**GG**	0.7412	0.7412	Additive	0.7352	0.7352	1.02 (0.9-1.16)	Additive	0.7789	0.7789	1.02 (0.89-1.17)
Case	1705 (76.53%)	659 (59.16%)	387 (34.74%)	68 (6.1%)			CC + CG vs GG	0.3930	0.393	0.86 (0.62-1.21)	CC + CG vs GG	0.3078	0.3078	0.83 (0.59-1.18)
Control	2177 (76.12%)	823 (57.55%)	531 (37.13%)	76 (5.31%)			CC vs CG + GG	0.4161	0.5239	1.07 (0.91-1.25)	CC vs CG + GG	0.4124	0.5727	1.07 (0.91-1.26)

^∗^ *p*_trend_: the *p* value from the Cochran-Armitage trend test with 100000 permutations.

**Table 2:** Association of *RANK*, *RANKL*, and *OPG* SNPs with syndesmophyte formation in Taiwanese AS patients.

SNP	Risk allele frequency	Genotype frequency (%)			*p*_trend_^∗^	*p*_FDR_	Test for the mode of inheritance unadjusted				Test for the mode of inheritance adjusted for sex			
								*p*	*p*_FDR_	OR (95% CI)		*p*	*p*_FDR_	OR (95% CI)
RANK rs1805034 CT	T	**CC**	**CT**	**TT**			Additive	0.4892	0.7257	1.07 (0.89-1.27)	Additive	0.4265	0.5118	1.08 (0.9-1.29)
Syndesmophyte+	655 (67.53%)	53 (10.93%)	209 (43.09%)	223 (45.98%)	0.4908	0.7362	TT + CT vs CC	0.9067	0.9067	0.98 (0.67-1.43)	TT + CT vs CC	0.9552	0.9552	1.01 (0.69-1.49)
Syndesmophyte-	840 (66.14%)	68 (10.71%)	294 (46.3%)	273 (42.99%)			TT vs CT + CC	0.3187	0.4781	1.13 (0.89-1.43)	TT vs CT + CC	0.3042	0.4564	1.14 (0.89-1.45)
	C	**CC**	**CT**	**TT**			Additive	0.3831	0.5746	1.07 (0.92-1.25)	Additive	0.2630	0.3946	1.1 (0.93-1.3)
Syndesmophyte+	315 (32.47%)	53 (10.93%)	209 (43.09%)	223 (45.98%)	0.4009	0.6014	CC + CT vs TT	0.4265	0.6398	1.09 (0.89-1.34)	CC + CT vs TT	0.3437	0.4173	1.11 (0.89-1.38)
Normal	881 (30.96%)	142 (9.98%)	597 (41.95%)	684 (48.07%)			CC vs CT + TT	0.5514	0.6617	1.11 (0.79-1.54)	CC vs CT + TT	0.3769	0.5557	1.18 (0.82-1.68)

RANKL rs7984870 CG	G	**CC**	**CG**	**GG**	0.0976	0.1952	Additive	0.0891	0.1781	1.16 (0.98-1.37)	Additive	0.0724	0.1629	1.17 (0.99-1.39)
Syndesmophyte+	542 (55.99%)	92 (19.01%)	242 (50%)	150 (30.99%)			GG + CG vs CC	0.1385	0.296	1.25 (0.93-1.67)	GG + CG vs CC	0.0975	0.2494	1.29 (0.96-1.74)
Syndesmophyte-	661 (52.38%)	143 (22.66%)	315 (49.92%)	173 (27.42%)			GG vs CG + CC	0.1923	0.3847	1.19 (0.92-1.54)	GG vs CG + CC	0.1951	0.4148	1.19 (0.91-1.56)
	G	**CC**	**CG**	**GG**			Additive	0.0080	0.0164	1.22 (1.05-1.41)	Additive	0.0141	0.0351	1.21 (1.04-1.42)
Syndesmophyte+	542 (55.99%)	92 (19.01%)	242 (50%)	150 (30.99%)	0.0083	0.022	GG + CG vs CC	0.0209	0.0563	1.35 (1.05-1.75)	GG + CG vs CC	0.0120	**0.0325**	1.42 (1.08-1.86)
Normal	1456 (51.05%)	344 (24.12%)	708 (49.65%)	374 (26.23%)			GG vs CG + CC	0.0426	0.0852	1.26 (1.01-1.58)	GG vs CG + CC	0.1222	0.2468	1.21 (0.95-1.54)

RANKL rs9525641 CT	T	**CC**	**CT**	**TT**			Additive	0.0776	0.1781	1.16 (0.98-1.38)	Additive	0.0814	0.1629	1.17 (0.98-1.39)
Syndesmophyte+	542 (56.22%)	92 (19.09%)	238 (49.38%)	152 (31.54%)	0.074	0.1952	TT + CT vs CC	0.1480	0.296	1.24 (0.93-1.66)	TT + CT vs CC	0.1247	0.2494	1.27 (0.94-1.71)
Syndesmophyte-	662 (52.46%)	143 (22.66%)	314 (49.76%)	174 (27.58%)			TT vs CT + CC	0.1506	0.3847	1.21 (0.93-1.57)	TT vs CT + CC	0.1851	0.4148	1.2 (0.92-1.56)
	T	**CC**	**CT**	**TT**			Additive	0.0054	0.0164	1.23 (1.06-1.43)	Additive	0.0118	0.0351	1.22 (1.05-1.42)
Syndesmophyte+	542 (56.22%)	92 (19.09%)	238 (49.38%)	152 (31.54%)	0.0064	0.022	TT + CT vs CC	0.0209	0.0563	1.35 (1.05-1.75)	TT + CT vs CC	0.0141	**0.0325**	1.4 (1.07-1.84)
Normal	1458 (51.01%)	346 (24.21%)	708 (49.55%)	375 (26.24%)			TT vs CT + CC	0.0247	0.0852	1.29 (1.03-1.62)	TT vs CT + CC	0.0888	0.2468	1.23 (0.97-1.56)

RANKL rs9533155 GC	C	**GG**	**GC**	**CC**	0.0825	0.1952	Additive	0.0809	0.1781	1.16 (0.98-1.38)	Additive	0.0675	0.1629	1.18 (0.99-1.4)
Syndesmophyte+	559 (57.75%)	85 (17.56%)	239 (49.38%)	160 (33.06%)			CC + GC vs GG	0.1264	0.296	1.27 (0.94-1.71)	CC + GC vs GG	0.0772	0.2494	1.32 (0.97-1.8)
Syndesmophyte-	682 (54.04%)	134 (21.24%)	312 (49.45%)	185 (29.32%)			CC vs GC + GG	0.1808	0.3847	1.19 (0.92-1.53)	CC vs GC + GG	0.2074	0.4148	1.18 (0.91-1.54)
	C	**GG**	**GC**	**CC**			Additive	0.0082	0.0164	1.22 (1.05-1.41)	Additive	0.0175	0.0351	1.21 (1.03-1.41)
Syndesmophyte+	559 (57.75%)	85 (17.56%)	239 (49.38%)	160 (33.06%)	0.011	0.022	CC + GC vs GG	0.0282	0.0563	1.35 (1.03-1.75)	CC + GC vs GG	0.0163	**0.0325**	1.41 (1.07-1.86)
Normal	1508 (52.84%)	318 (22.28%)	710 (49.75%)	399 (27.96%)			CC vs GC + GG	0.0334	0.0852	1.27 (1.02-1.59)	CC vs GC + GG	0.1234	0.2468	1.2 (0.95-1.52)

OPG rs3102735 CT	T	**CC**	**CT**	**TT**	0.8545	0.8545	Additive	0.8152	0.8152	1.03 (0.81-1.31)	Additive	0.9277	0.9277	0.99 (0.77-1.26)
Syndesmophyte+	824 (85.48%)	12 (2.49%)	116 (24.07%)	354 (73.44%)			TT + CT vs CC	0.2008	0.3012	0.57 (0.24-1.35)	TT + CT vs CC	0.1698	0.2546	0.53 (0.21-1.31)
Syndesmophyte-	1076 (85.13%)	9 (1.42%)	170 (26.9%)	453 (71.68%)			TT vs CT + CC	0.5145	0.6174	1.09 (0.84-1.42)	TT vs CT + CC	0.7467	0.7467	1.05 (0.8-1.37)
	C	**CC**	**CT**	**TT**			Additive	0.7260	0.726	1.04 (0.85-1.27)	Additive	0.4985	0.5982	1.08 (0.87-1.34)
Syndesmophyte+	140 (14.52%)	12 (2.49%)	116 (24.07%)	354 (73.44%)	0.7497	0.7497	CC + CT vs TT	0.7689	0.7689	1.04 (0.82-1.31)	CC + CT vs TT	0.5220	0.522	1.09 (0.85-1.39)
Normal	400 (14.06%)	32 (2.25%)	336 (23.63%)	1054 (74.12%)			CC vs CT + TT	0.7626	0.7626	1.11 (0.57-2.16)	CC vs CT + TT	0.7035	0.7035	1.15 (0.56-2.36)

OPG rs2073618 CG	C	**CC**	**CG**	**GG**	0.6146	0.7375	Additive	0.6048	0.7257	1.05 (0.87-1.28)	Additive	0.3549	0.5118	1.1 (0.9-1.34)
Syndesmophyte+	746 (77.07%)	290 (59.92%)	166 (34.3%)	28 (5.79%)			CC + CG vs GG	0.6986	0.8383	1.1 (0.67-1.81)	CC + CG vs GG	0.4582	0.5498	1.21 (0.73-2)
Syndesmophyte-	959 (76.11%)	369 (58.57%)	221 (35.08%)	40 (6.35%)			CC vs CG + GG	0.6508	0.6508	1.06 (0.83-1.34)	CC vs CG + GG	0.4325	0.519	1.1 (0.86-1.41)
	C	**CC**	**CG**	**GG**			Additive	0.5471	0.6565	1.05 (0.89-1.25)	Additive	0.7931	0.7931	1.03 (0.85-1.23)
Syndesmophyte+	746 (77.07%)	290 (59.92%)	166 (34.3%)	28 (5.79%)	0.5674	0.6809	CC + CG vs GG	0.6932	0.7689	0.91 (0.59-1.42)	CC + CG vs GG	0.3477	0.4173	0.79 (0.49-1.28)
Normal	2177 (76.12%)	823 (57.55%)	531 (37.13%)	76 (5.31%)			CC vs CG + GG	0.3621	0.5431	1.1 (0.89-1.36)	CC vs CG + GG	0.4631	0.5557	1.09 (0.87-1.36)

^∗^ *p*_trend_: the *p* value from the Cochran-Armitage trend test with 100000 permutations.

**Table 3:** Association of *RANK*, *RANKL*, and *OPG* SNPs with HLA-B27 in Taiwanese AS patients.

SNP	Risk allele frequency	Genotype frequency (%)			*p*_trend_^∗^	*p*_FDR_	Test for mode of inheritance unadjusted				Test for mode of inheritance adjusted for sex			
								*p*	*p*_FDR_	OR (95% CI)		*p*	*p*_FDR_	OR (95% CI)
RANK rs1805034 CT	T	**CC**	**CT**	**TT**			Additive	0.1468	0.4113	1.25 (0.93-1.69)	Additive	0.1315	0.3764	1.27 (0.93-1.72)
B27 positive	1372 (67.19%)	109 (10.68%)	452 (44.27%)	460 (45.05%)	0.1497	0.4491	TT + CT vs CC	0.6584	0.98	1.15 (0.61-2.17)	TT + CT vs CC	0.5913	0.9876	1.19 (0.63-2.25)
B27 negative	123 (62.12%)	12 (12.12%)	51 (51.52%)	36 (36.36%)			TT vs CT + CC	0.0980	0.2007	1.43 (0.94-2.2)	TT vs CT + CC	0.0954	0.2161	1.44 (0.94-2.21)
	C	**CC**	**CT**	**TT**			Additive	0.1714	0.2571	1.09 (0.96-1.23)	Additive	0.2097	0.2587	1.08 (0.96-1.23)
B27 positive	670 (32.81%)	109 (10.68%)	452 (44.27%)	460 (45.05%)	0.1773	0.266	CC + CT vs TT	0.1409	0.2114	1.13 (0.96-1.33)	CC + CT vs TT	0.1636	0.1963	1.13 (0.95-1.33)
Normal	881 (30.96%)	142 (9.98%)	597 (41.95%)	684 (48.07%)			CC vs CT + TT	0.5740	0.574	1.08 (0.83-1.4)	CC vs CT + TT	0.6572	0.6572	1.06 (0.81-1.4)

RANKL rs7984870 CG	G	**CC**	**CG**	**GG**	0.3311	0.4512	Additive	0.3080	0.4113	1.16 (0.87-1.56)	Additive	0.2904	0.3764	1.17 (0.87-1.58)
B27 positive	1103 (54.28%)	214 (21.06%)	501 (49.31%)	301 (29.63%)			GG + CG vs CC	0.9722	0.98	1.01 (0.61-1.67)	GG + CG vs CC	0.9153	0.9876	1.03 (0.62-1.7)
B27 negative	100 (50.51%)	21 (21.21%)	56 (56.57%)	22 (22.22%)			GG vs CG + CC	0.1230	0.2007	1.47 (0.9-2.41)	GG vs CG + CC	0.1253	0.2161	1.47 (0.9-2.41)
	G	**CC**	**CG**	**GG**			Additive	0.0266	0.0637	1.14 (1.02-1.27)	Additive	0.0386	0.1003	1.13 (1.01-1.28)
B27 positive	1103 (54.28%)	214 (21.06%)	501 (49.31%)	301 (29.63%)	0.0269	0.0638	GG + CG vs CC	0.0760	0.2114	1.19 (0.98-1.44)	GG + CG vs CC	0.0585	0.1715	1.22 (0.99-1.49)
Normal	1456 (51.05%)	344 (24.12%)	708 (49.65%)	374 (26.23%)			GG vs CG + CC	0.0643	0.1286	1.18 (0.99-1.42)	GG vs CG + CC	0.1321	0.2666	1.16 (0.96-1.39)

RANKL rs9525641 CT	T	**CC**	**CT**	**TT**	0.376	0.4512	Additive	0.3632	0.4113	1.15 (0.86-1.53)	Additive	0.3764	0.3764	1.14 (0.85-1.53)
B27 positive	1103 (54.39%)	214 (21.1%)	497 (49.01%)	303 (29.88%)			TT + CT vs CC	0.9800	0.98	1.01 (0.61-1.66)	TT + CT vs CC	0.9591	0.9876	1.01 (0.61-1.68)
B27 negative	101 (51.01%)	21 (21.21%)	55 (55.56%)	23 (23.23%)			TT vs CT + CC	0.1672	0.2007	1.41 (0.87-2.28)	TT vs CT + CC	0.1867	0.224	1.39 (0.85-2.26)
	T	**CC**	**CT**	**TT**			Additive	0.0208	0.0637	1.14 (1.02-1.28)	Additive	0.0322	0.1003	1.14 (1.01-1.28)
B27 positive	1103 (54.39%)	214 (21.1%)	497 (49.01%)	303 (29.88%)	0.0206	0.0638	TT + CT vs CC	0.0719	0.2114	1.19 (0.99-1.45)	TT + CT vs CC	0.0529	0.1715	1.22 (1-1.49)
Normal	1458 (51.01%)	346 (24.21%)	708 (49.55%)	375 (26.24%)			TT vs CT + CC	0.0479	0.1286	1.2 (1-1.43)	TT vs CT + CC	0.1132	0.2666	1.16 (0.97-1.4)

RANKL rs9533155 GC	C	**GG**	**GC**	**CC**	0.3659	0.4512	Additive	0.3539	0.4113	1.15 (0.86-1.54)	Additive	0.3372	0.3764	1.16 (0.86-1.55)
B27 positive	1137 (55.95%)	200 (19.69%)	495 (48.72%)	321 (31.59%)			CC + GC vs GG	0.9069	0.98	0.97 (0.58-1.63)	CC + GC vs GG	0.9876	0.9876	1 (0.59-1.68)
B27 negative	104 (52.53%)	19 (19.19%)	56 (56.57%)	24 (24.24%)			CC vs GC + GG	0.1329	0.2007	1.44 (0.9-2.32)	CC vs GC + GG	0.1441	0.2161	1.43 (0.89-2.31)
	C	**GG**	**GC**	**CC**			Additive	0.0319	0.0637	1.13 (1.01-1.27)	Additive	0.0502	0.1003	1.13 (1-1.27)
B27 positive	1137 (55.95%)	200 (19.69%)	495 (48.72%)	321 (31.59%)	0.0319	0.0638	CC + GC vs GG	0.1215	0.2114	1.17 (0.96-1.43)	CC + GC vs GG	0.0858	0.1715	1.2 (0.98-1.48)
Normal	1508 (52.84%)	318 (22.28%)	710 (49.75%)	399 (27.96%)			CC vs GC + GG	0.0523	0.1286	1.19 (1-1.42)	CC vs GC + GG	0.1333	0.2666	1.15 (0.96-1.38)

OPG rs3102735 CT	C	**CC**	**CT**	**TT**	0.4528	0.4528	Additive	0.4113	0.4113	1.2 (0.78-1.87)	Additive	0.3280	0.3764	1.25 (0.8-1.95)
B27 positive	303 (14.91%)	19 (1.87%)	265 (26.08%)	732 (72.05%)			CC + CT vs TT	0.3438	0.98	1.27 (0.78-2.05)	CC + CT vs TT	0.2662	0.7985	1.32 (0.81-2.15)
B27 negative	25 (12.76%)	2 (2.04%)	21 (21.43%)	75 (76.53%)			CC vs CT + TT	0.9055	0.9055	0.91 (0.21-3.96)	CC vs CT + TT	0.9262	0.9262	0.93 (0.21-4.07)
	C	**CC**	**CT**	**TT**			Additive	0.4052	0.4416	1.07 (0.91-1.26)	Additive	0.2156	0.2587	1.11 (0.94-1.32)
B27 positive	303 (14.91%)	19 (1.87%)	265 (26.08%)	732 (72.05%)	0.4048	0.446	CC + CT vs TT	0.2542	0.305	1.11 (0.93-1.33)	CC + CT vs TT	0.1167	0.1751	1.17 (0.96-1.41)
Normal	400 (14.06%)	32 (2.25%)	336 (23.63%)	1054 (74.12%)			CC vs CT + TT	0.5183	0.574	0.83 (0.47-1.46)	CC vs CT + TT	0.5798	0.6572	0.84 (0.46-1.53)

OPG rs2073618 CG	C	**CC**	**CG**	**GG**	0.0645	0.387	Additive	0.0575	0.3448	1.36 (0.99-1.88)	Additive	0.0333	0.1999	1.42 (1.03-1.96)
B27 positive	1566 (77.07%)	608 (59.84%)	350 (34.45%)	58 (5.71%)			CC + CG vs GG	0.0803	0.482	1.88 (0.93-3.79)	CC + CG vs GG	0.0481	0.2888	2.05 (1.01-4.18)
B27 negative	139 (70.92%)	51 (52.04%)	37 (37.76%)	10 (10.2%)			CC vs CG + GG	0.1347	0.2007	1.37 (0.91-2.08)	CC vs CG + GG	0.0956	0.2161	1.43 (0.94-2.17)
	C	**CC**	**CG**	**GG**			Additive	0.4416	0.4416	1.05 (0.92-1.21)	Additive	0.4730	0.473	1.05 (0.91-1.21)
B27 positive	1566 (77.07%)	608 (59.84%)	350 (34.45%)	58 (5.71%)	0.446	0.446	CC + CG vs GG	0.6715	0.6715	0.93 (0.65-1.31)	CC + CG vs GG	0.4772	0.4772	0.87 (0.6-1.26)
Normal	2177 (76.12%)	823 (57.55%)	531 (37.13%)	76 (5.31%)			CC vs CG + GG	0.2574	0.386	1.1 (0.93-1.29)	CC vs CG + GG	0.2314	0.3471	1.11 (0.94-1.32)

^∗^ *p*_trend_: the *p* value from the Cochran-Armitage trend test with 100000 permutations.

**Table 4:** Association of RANKL SNP haplotypes with AS susceptibility in Taiwanese.

Haplotypes of rs7984870, rs9533155, and rs9525641	Estimated frequency (%)			Permutation	Logistic regression		Logistic regression adjusted for sex	
	AS (*N* = 1120)	Normal (*N* = 1435)	All (*N* = 2555)	*p* value^∗^	*p* value	OR (95% CI)	*p* value	OR (95% CI)
G-C-T	51.41%	49.74%	50.48%	0.2412	0.2224	1.07 (0.96-1.2)	0.2620	1.07 (0.95-1.2)
C-G-C	41.83%	45.88%	44.10%	0.0034	0.0042	0.85 (0.76-0.95)	**0.0081**	0.85 (0.76-0.96)
Others	6.76%	4.38%	5.42%		3.33 × 10^−4^	1.57 (1.23-2)	9.06 × 10^−4^	1.54 (1.19-2)

^∗^The *p* value for the estimated haplotype was generated from 10000 permutations using the EM algorithm.

## Data Availability

The data used to support the findings of this study are available from the corresponding author upon request.

## Supplementary Materials

Supplementary Materials Table S1: association of the *RANKL/RANK/OPG* SNPs with age of disease onset in the AS patients. Table S2: haplotype association of the *RANKL* SNPs rs7984870, rs9533155, and rs9525641 with syndesmophyte formation in AS cases. Table S3: haplotype association of the *RANKL* SNPs rs7984870, rs9533155, and rs9525641 with HLA-B27 in AS cases. Table S4: haplotype association of the *RANKL* SNPs rs7984870, rs9533155, and rs9525641 with age of disease onset in AS cases. Table S5: haplotype association of the *OPG* SNPs rs2073618 and rs3102735 in the AS cases and healthy controls. Table S6: haplotype association of the *OPG* SNPs rs2073618 and rs3102735 with syndesmophyte formation in the AS cases. Table S7: haplotype association of the *OPG* SNPs rs2073618 and rs3102735 with age of disease onset in the AS cases. Table S8: haplotype association of the *OPG* SNPs rs2073618 and rs3102735 with HLA-B27 in the AS cases. Figure S1: the promoter activity by transient transfection of hFOB 1.19 cells with plasmid constructs that contained different *RANKL* promoters and a luciferase reporter.
