# Supplementary material for: Association of Genetic Variants of RANK, RANKL, and OPG with Ankylosing Spondylitis Clinical Features in Taiwanese
Source: Mediators Inflamm. 2019 Mar 20;2019:8029863. doi: 10.1155/2019/8029863 (PMC6446096; doi:10.1155/2019/8029863)

Association of genetic variants of *RANK*, *RANKL*, and *OPG* with ankylosing spondylitis clinical features in Taiwanese

Chin-Man Wang[1], Shu-Chun Tsai[2] Jing-Chi Lin[3], Yeong-Jian Jan Wu[3], Jianming Wu[4], Ji-Yih Chen [3]

Supplemental Table 1 Association of the *RANKL/RANK/OPG* SNPs with age of disease onset in the AS patients

**SNP**	**Risk Allele Frequency**	**Genotype Frequency (%)**			**P_{Trend}***	**P_{FDR}**	**Test for mode of inheritance unadjusted**				**Test for mode of inheritance adjusted for sex**			
								**P**	**P_{FDR}**	**OR (95% CI)**		**P**	**P_{FDR}**	**OR (95% CI)**
**RANKL rs1805034**														
**CT**	C	**CC**	**CT**	**TT**			Additive	0.4471	0.5365	1.08 (0.89-1.32)	Additive	0.4673	0.6131	1.08 (0.88-1.31)
age group≦16	205 (34.51%)	29 (9.76%)	147 (49.49%)	121 (40.74%)	0.478	0.5736	CC+CT vs TT	0.1516	0.4299	1.22 (0.93-1.59)	CC+CT vs TT	0.1500	0.4389	1.22 (0.93-1.6)
age group＞16	540 (32.81%)	92 (11.18%)	356 (43.26%)	375 (45.57%)			CC vs CT+TT	0.5012	0.6835	0.86 (0.55-1.33)	CC vs CT+TT	0.4546	0.5625	0.84 (0.54-1.31)
	C	**CC**	**CT**	**TT**			Additive	0.0900	0.5402	1.18 (0.98-1.42)	Additive	0.0880	0.5282	1.19 (0.98-1.44)
age group≦16	205 (34.51%)	29 (9.76%)	147 (49.49%)	121 (40.74%)	0.0965	0.579	CC+CT vs TT	0.0216	0.1298	1.35 (1.05-1.73)	CC+CT vs TT	0.0290	0.1742	1.34 (1.03-1.74)
Normal	881 (30.96%)	142 (9.98%)	597 (41.95%)	684 (48.07%)			CC vs CT+TT	0.9110	0.9391	0.98 (0.64-1.48)	CC vs CT+TT	0.9358	0.9745	1.02 (0.66-1.57)
**RANKL rs7984870**														
**CG**	C	**CC**	**CG**	**GG**	0.1811	0.5433	Additive	0.1662	0.4987	1.14 (0.95-1.38)	Additive	0.1717	0.5150	1.14 (0.95-1.38)
age group≦16	287 (48.48%)	71 (23.99%)	145 (48.99%)	80 (27.03%)			CC+CG vs GG	0.3904	0.4299	1.14 (0.85-1.53)	CC+CG vs GG	0.3782	0.4389	1.14 (0.85-1.54)
age group＞16	740 (45.18%)	164 (20.02%)	412 (50.31%)	243 (29.67%)			CC vs CG+GG	0.1525	0.4576	1.26 (0.92-1.73)	CC vs CG+GG	0.1687	0.5061	1.25 (0.91-1.72)
	G	**CC**	**CG**	**GG**			Additive	0.8364	0.9411	1.02 (0.85-1.21)	Additive	0.8342	0.9687	1.02 (0.85-1.22)
age group≦16	305 (51.52%)	71 (23.99%)	145 (48.99%)	80 (27.03%)	0.8553	0.9662	GG+CG vs CC	0.9601	0.9601	1.01 (0.75-1.35)	GG+CG vs CC	0.7817	0.9380	1.04 (0.77-1.41)
Normal	1456 (51.05%)	344 (24.12%)	708 (49.65%)	374 (26.23%)			GG vs CG+CC	0.7749	0.9391	1.04 (0.79-1.38)	GG vs CG+CC	0.9448	0.9745	1.01 (0.76-1.35)
**RANKL rs9525641**														
**CT**	C	**CC**	**CT**	**TT**	0.0996	0.5433	Additive	0.0983	0.4987	1.17 (0.97-1.41)	Additive	0.0914	0.5150	1.18 (0.97-1.42)
age group≦16	289 (48.82%)	73 (24.66%)	143 (48.31%)	80 (27.03%)			CC+CT vs TT	0.3182	0.4299	1.16 (0.87-1.56)	CC+CT vs TT	0.2862	0.4389	1.18 (0.87-1.58)
age group＞16	733 (44.86%)	162 (19.83%)	409 (50.06%)	246 (30.11%)			CC vs CT+TT	0.0814	0.4576	1.32 (0.97-1.81)	CC vs CT+TT	0.0839	0.5034	1.32 (0.96-1.81)
	T	**CC**	**CT**	**TT**			Additive	0.9411	0.9411	1.01 (0.85-1.2)	Additive	0.9687	0.9687	1 (0.84-1.2)
age group≦16	303 (51.18%)	73 (24.66%)	143 (48.31%)	80 (27.03%)	0.9662	0.9662	TT+CT vs CC	0.8691	0.9601	0.98 (0.73-1.3)	TT+CT vs CC	0.9741	0.9741	1.01 (0.74-1.36)
Normal	1458 (51.01%)	346 (24.21%)	708 (49.55%)	375 (26.24%)			TT vs CT+CC	0.7790	0.9391	1.04 (0.79-1.38)	TT vs CT+CC	0.9745	0.9745	1 (0.75-1.34)

**RANKL rs9533155 GC**	G	**GG**	**GC**	**CC**	0.2868	0.5736	Additive	0.2690	0.5365	1.11 (0.92-1.34)	Additive	0.2753	0.5505	1.11 (0.92-1.34)
age group≦16	274 (46.28%)	64 (21.62%)	146 (49.32%)	86 (29.05%)			GG+GC vs CC	0.4125	0.4299	1.13 (0.85-1.51)	GG+GC vs CC	0.3837	0.4389	1.14 (0.85-1.52)
age group＞16	715 (43.65%)	155 (18.93%)	405 (49.45%)	259 (31.62%)			GG vs GC+CC	0.3173	0.6347	1.18 (0.85-1.64)	GG vs GC+CC	0.3594	0.5625	1.17 (0.84-1.62)
	C	**GG**	**GC**	**CC**			Additive	0.6973	0.9411	1.04 (0.87-1.24)	Additive	0.7039	0.9687	1.04 (0.86-1.24)
age group≦16	318 (53.72%)	64 (21.62%)	146 (49.32%)	86 (29.05%)	0.714	0.9662	CC+GC vs GG	0.8041	0.9601	1.04 (0.77-1.41)	CC+GC vs GG	0.6008	0.9013	1.09 (0.8-1.49)
Normal	1508 (52.84%)	318 (22.28%)	710 (49.75%)	399 (27.96%)			CC vs GC+GG	0.7035	0.9391	1.06 (0.8-1.39)	CC vs GC+GG	0.9077	0.9745	1.02 (0.77-1.35)
**OPG rs3102735 CT**	T	**CC**	**CT**	**TT**	0.7876	0.7876	Additive	0.7379	0.7379	1.05 (0.8-1.37)	Additive	0.8411	0.8411	1.03 (0.78-1.35)
age group≦16	509 (85.69%)	4 (1.35%)	77 (25.93%)	216 (72.73%)			TT+CT vs CC	0.4299	0.4299	1.56 (0.52-4.63)	TT+CT vs CC	0.4389	0.4389	1.55 (0.52-4.62)
age group＞16	1391 (85.13%)	17 (2.08%)	209 (25.58%)	591 (72.34%)			TT vs CT+CC	0.8979	0.8979	1.02 (0.76-1.37)	TT vs CT+CC	0.9879	0.9879	1 (0.74-1.34)
	C	**CC**	**CT**	**TT**			Additive	0.8759	0.9411	1.02 (0.79-1.31)	Additive	0.7195	0.9687	1.05 (0.81-1.36)
age group≦16	85 (14.31%)	4 (1.35%)	77 (25.93%)	216 (72.73%)	0.8955	0.9662	CC+CT vs TT	0.6190	0.9601	1.07 (0.81-1.42)	CC+CT vs TT	0.4786	0.9013	1.11 (0.83-1.49)
Normal	400 (14.06%)	32 (2.25%)	336 (23.63%)	1054 (74.12%)			CC vs CT+TT	0.3280	0.9391	0.59 (0.21-1.68)	CC vs CT+TT	0.3492	0.9745	0.6 (0.21-1.74)
**OPG rs2073618 CG**	G	**CC**	**CG**	**GG**	0.4029	0.5736	Additive	0.4031	0.5365	1.1 (0.88-1.36)	Additive	0.5109	0.6131	1.08 (0.87-1.33)
age group≦16	146 (24.75%)	165 (55.93%)	114 (38.64%)	16 (5.42%)			GG+CG vs CC	0.1892	0.4299	1.2 (0.92-1.57)	GG+CG vs CC	0.2405	0.4389	1.18 (0.9-1.54)
age group＞16	377 (23.02%)	494 (60.32%)	273 (33.33%)	52 (6.35%)			GG vs CG+CC	0.5696	0.6835	0.85 (0.48-1.5)	GG vs CG+CC	0.4687	0.5625	0.81 (0.45-1.44)
	G	**CC**	**CG**	**GG**			Additive	0.6505	0.9411	1.05 (0.85-1.29)	Additive	0.4477	0.9687	1.09 (0.88-1.35)
age group≦16	146 (24.75%)	165 (55.93%)	114 (38.64%)	16 (5.42%)	0.6743	0.9662	GG+CG vs CC	0.6085	0.9601	1.07 (0.83-1.37)	GG+CG vs CC	0.4867	0.9013	1.1 (0.85-1.42)
Normal	683 (23.88%)	823 (57.55%)	531 (37.13%)	76 (5.31%)			GG vs CG+CC	0.9391	0.9391	1.02 (0.59-1.77)	GG vs CG+CC	0.6306	0.9745	1.15 (0.65-2.05)

*PTrend: the p-value from Cochran–Armitage trend test with 100,000 permutations.

Supplemental Table 2 Haplotype association of the *RANKL* SNPs rs7984870, rs9533155 and rs9525641 with syndesmophyte formation in AS cases

**Haplotype of rs7984870,rs9533155 and rs9525641**	**Estimated Frequency (%)**			**Permutation**	**Logistic regression**		**Logistic regression adjusted for sex**	
	**Syndesmophyte+** (N=485)	**Syndesmophyte-** (N=635)	**AS cases** (N=1120)	**P value***	**P value**	**OR (95% CI)**	**P value**	**OR (95% CI)**
G-C-T	53.34%	49.94%	51.41%	0.1135	0.1103	1.15 (0.97-1.36)	0.0990	1.16 (0.97-1.37)
C-G-C	39.71%	43.45%	41.83%	0.0713	0.0760	0.86 (0.72-1.02)	**0.0724**	0.85 (0.72-1.01)
Others	6.95%	6.60%	6.76%		0.7549	1.05 (0.75-1.48)	0.8037	1.04 (0.74-1.47)

Note*: The p-value for the estimated haplotype was generated from 10,000 permutations using the EM algorithm.

Supplemental Table 3 Haplotype association of the *RANKL* SNPs rs7984870, rs9533155 and rs9525641 with HLA-B27 in AS cases

**Haplotype of rs7984870,rs9533155 and rs9525641**	**Estimated Frequency (%)**			**Permutation**	**Logistic regression**		**Logistic regression adjusted for sex**	
	**B27 positive** (N=1021)	**B27 negative** (N=99)	**AS cases** (N=1120)	**P value***	**P value**	**OR (95% CI)**	**P value**	**OR (95% CI)**
G-C-T	51.70%	48.43%	51.41%	0.3959	0.3775	1.14 (0.85-1.53)	0.3717	1.14 (0.85-1.53)
C-G-C	41.48%	45.40%	41.83%	0.2788	0.2874	0.85 (0.64-1.14)	0.2900	0.85 (0.63-1.15)
Others	6.82%	6.16%	6.76%		0.7356	1.11 (0.6-2.04)	0.7599	1.1 (0.6-2.03)

Note*: The p-value for the estimated haplotype was generated from 10,000 permutations using the EM algorithm.

Supplemental Table 4 Haplotype association of the *RANKL* SNPs rs7984870, rs9533155 and rs9525641 with age of disease onset in AS cases

**Haplotype of rs7984870,rs9533155 and rs9525641**	**Estimated Frequency (%)**			**Permutation**	**Logistic regression**		**Logistic regression adjusted for sex**	
	**age group≦16** (N=297)	**age group > 16** (N=823)	**AS cases** (N=1120)	**P value***	**P value**	**OR (95% CI)**	**P value**	**OR (95% CI)**
G-C-T	49.63%	52.06%	51.41%	0.3206	0.3015	0.91 (0.75-1.09)	0.3011	0.91 (0.75-1.09)
C-G-C	44.91%	40.71%	41.83%	0.0718	0.0779	1.19 (0.98-1.43)	**0.0748**	1.19 (0.98-1.44)
Others	5.47%	7.23%	6.76%		0.1564	0.75 (0.5-1.12)	0.1468	0.74 (0.49-1.11)

Note*: The p-value for the estimated haplotype was generated from 10,000 permutations using the EM algorithm.

Supplemental Table 5 Haplotype association of the *OPG* SNPs rs2073618 and rs3102735 in the AS cases and healthy controls

**Haplotype of rs2073618 and rs3102735**	**Estimated Frequency (%)**			**Permutation**	**Logistic regression**		**Logistic regression adjusted for sex**	
	**AS** (N=1120)	**Normal** (N=1435)	**All** (N=2555)	**P value***	**P value**	**OR (95% CI)**	**P value**	**OR (95% CI)**
C-T	62.37%	62.87%	62.63%	0.7237	0.7969	0.99 (0.88-1.1)	0.5323	0.96 (0.85-1.08)
G-T	22.90%	23.08%	23.02%	0.8861	0.7485	0.98 (0.86-1.12)	0.7935	0.98 (0.86-1.13)
C-C	14.15%	13.27%	13.67%	0.3710	0.4397	1.06 (0.91-1.25)	0.2256	1.11 (0.94-1.31)
G-C	0.58%	0.79%	0.68%	0.5721	0.8829	0.92 (0.29-2.89)	0.8923	0.92 (0.28-3.05)

Note*: The p-value for the estimated haplotype was generated from 10,000 permutations using the EM algorithm.

Supplemental Table 6 Haplotype association of the *OPG* SNPs rs2073618 and rs3102735 with syndesmophyte formation in the AS cases

Haplotype of rs2073618 and rs3102735	Estimated Frequency (%)			Permutation	Logistic regression		Logistic regression adjusted for sex	
	Syndesmophyte+ (N=485)	Syndesmophyte- (N=635)	AS cases (N=1120)	P value*	P value	OR (95% CI)	P value	OR (95% CI)
C-T	63.47%	61.45%	62.37%	0.3411	0.4510	1.07 (0.9-1.27)	0.3719	1.08 (0.91-1.29)
G-T	22.00%	23.67%	22.90%	0.3649	0.5151	0.94 (0.77-1.14)	0.2854	0.89 (0.73-1.1)
C-C	13.60%	14.65%	14.15%	0.4985	0.6320	0.94 (0.74-1.2)	0.8861	0.98 (0.77-1.26)
G-C	0.93%	0.24%	0.58%	0.1633	0.1384	5.25 (0.59-47.04)	0.1943	4.27 (0.48-38.31)

Note*: The p-value for the estimated haplotype was generated from 10,000 permutations using the EM algorithm.

Supplemental Table 7 Haplotype association of the *OPG* SNPs rs2073618 and rs3102735 with age of disease onset in the AS cases

**Haplotype of rs2073618 and rs3102735**	**Estimated Frequency (%)**			**Permutation**	**Logistic regression**		**Logistic regression adjusted for sex**	
	**age group≦16** (N=297)	**age group > 16** (N=823)	**AS cases** (N=1120)	**P value***	**P value**	**OR (95% CI)**	**P value**	**OR (95% CI)**
C-T	62.24%	62.51%	62.37%	0.8961	0.6882	0.96 (0.79-1.17)	0.7245	0.97 (0.8-1.17)
G-T	23.45%	22.61%	22.90%	0.6885	0.4770	1.08 (0.87-1.35)	0.5860	1.06 (0.85-1.33)
C-C	12.99%	14.48%	14.15%	0.3736	0.6947	0.95 (0.72-1.24)	0.8040	0.97 (0.74-1.27)
G-C	1.32%	0.41%	0.58%	0.0636	0.5030	1.84 (0.31-11.06)	0.5741	1.67 (0.28-10.03)

Note*: The p-value for the estimated haplotype was generated from 10,000 permutations using the EM algorithm.

Supplemental Table 8 Haplotype association of the *OPG* SNPs rs2073618 and rs3102735 with HLA-B27 in the AS cases

Haplotype of rs2073618 and rs3102735	Estimated Frequency (%)			Permutation	Logistic regression		Logistic regression adjusted for sex	
	B27 positive (N=1021)	B27 negative (N=99)	AS cases (N=1120)	P value*	P value	OR (95% CI)	P value	OR (95% CI)
C-T	62.68%	59.01%	62.37%	0.3228	0.2965	1.17 (0.87-1.58)	0.2679	1.18 (0.88-1.6)
G-T	22.40%	28.24%	22.90%	0.0763	0.0621	0.73 (0.53-1.02)	**0.0373**	0.7 (0.51-0.98)
C-C	14.38%	11.91%	14.15%	0.3554	0.3572	1.23 (0.79-1.92)	0.2784	1.28 (0.82-2)
G-C	0.54%	0.84%	0.58%	0.9340	0.3920	0.38 (0.04-3.44)	0.3037	0.32 (0.04-2.84)

Note*: The p-value for the estimated haplotype was generated from 10,000 permutations using the EM algorithm.

Supplemental Figure 1: The promoter activity by transient transfection of hFOB 1.19 cells with plasmid constructs that contained different *RANKL* promoters and a luciferase reporter. All the three reporter constructs (CTT, CTC, GCC) demonstrated much lower luciferase activities than the control vector, pGL

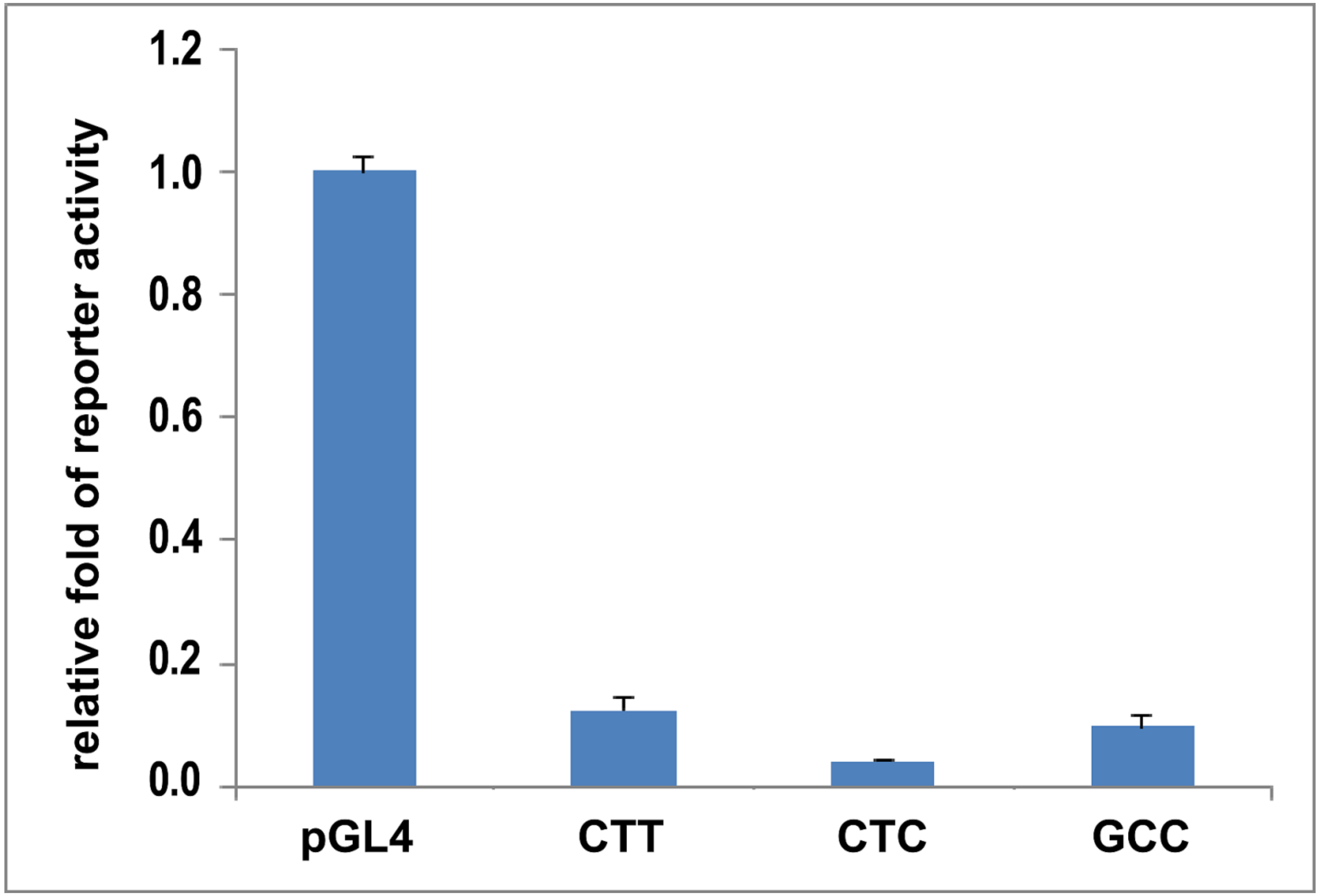

Supplement: Supplementary Materials — Table S1: association of the RANKL/RANK/OPG SNPs with age of disease onset in the AS patients. Table S2: haplotype association of the RANKL SNPs rs7984870, rs9533155, and rs9525641 with syndesmophyte formation in AS cases. Table S3: haplotype association of the RANKL SNPs rs7984870, rs9533155, and rs9525641 with HLA-B27 in AS cases. Table S4: haplotype association of the RANKL SNPs rs7984870, rs9533155, and rs9525641 with age of disease onset in AS cases. Table S5: haplotype association of the OPG SNPs rs2073618 and rs3102735 in the AS cases and healthy controls. Table S6: haplotype association of the OPG SNPs rs2073618 and rs3102735 with syndesmophyte formation in the AS cases. Table S7: haplotype association of the OPG SNPs rs2073618 and rs3102735 with age of disease onset in the AS cases. Table S8: haplotype association of the OPG SNPs rs2073618 and rs3102735 with HLA-B27 in the AS cases. Figure S1: the promoter activity by transient transfection of hFOB 1.19 cells with plasmid constructs that contained different RANKL promoters and a luciferase reporter. [file 8029863.f1.pdf]
